# GRAF1 deficiency blunts sarcolemmal injury repair and exacerbates cardiac and skeletal muscle pathology in dystrophin-deficient mice

**DOI:** 10.1186/s13395-015-0054-6

**Published:** 2015-08-21

**Authors:** Kaitlin C. Lenhart, Thomas J. O’Neill, Zhaokang Cheng, Rachel Dee, Alexis R. Demonbreun, Jianbin Li, Xiao Xiao, Elizabeth M. McNally, Christopher P. Mack, Joan M. Taylor

**Affiliations:** Department of Pathology and Laboratory Medicine, University of North Carolina at Chapel Hill, Chapel Hill, NC 27599 USA; Center for Genetic Medicine, Northwestern University Feinberg School of Medicine, Chicago, IL 60611 USA; Department of Gene Therapy Molecular Pharmaceutics, Northwestern University Feinberg School of Medicine, Chicago, IL USA; McAllister Heart Institute, University of North Carolina at Chapel Hill, Chapel Hill, NC 27599 USA

**Keywords:** Muscle repair, Dysferlin, Muscular dystrophy, Rho-GAP

## Abstract

**Background:**

The plasma membranes of striated muscle cells are particularly susceptible to rupture as they endure significant mechanical stress and strain during muscle contraction, and studies have shown that defects in membrane repair can contribute to the progression of muscular dystrophy. The synaptotagmin-related protein, dysferlin, has been implicated in mediating rapid membrane repair through its ability to direct intracellular vesicles to sites of membrane injury. However, further work is required to identify the precise molecular mechanisms that govern dysferlin targeting and membrane repair. We previously showed that the bin–amphiphysin–Rvs (BAR)–pleckstrin homology (PH) domain containing Rho-GAP GTPase regulator associated with focal adhesion kinase-1 (GRAF1) was dynamically recruited to the tips of fusing myoblasts wherein it promoted membrane merging by facilitating ferlin-dependent capturing of intracellular vesicles. Because acute membrane repair responses involve similar vesicle trafficking complexes/events and because our prior studies in GRAF1-deficient tadpoles revealed a putative role for GRAF1 in maintaining muscle membrane integrity, we postulated that GRAF1 might also play an important role in facilitating dysferlin-dependent plasma membrane repair.

**Methods:**

We used an in vitro laser-injury model to test whether GRAF1 was necessary for efficient muscle membrane repair. We also generated dystrophin/GRAF1 doubledeficient mice by breeding *mdx* mice with GRAF1 hypomorphic mice. Evans blue dye uptake and extensive morphometric analyses were used to assess sarcolemmal integrity and related pathologies in cardiac and skeletal muscles isolated from these mice.

**Results:**

Herein, we show that GRAF1 is dynamically recruited to damaged skeletal and cardiac muscle plasma membranes and that GRAF1-depleted muscle cells have reduced membrane healing abilities. Moreover, we show that dystrophin depletion exacerbated muscle damage in GRAF1-deficient mice and that mice with dystrophin/GRAF1 double deficiency phenocopied the severe muscle pathologies observed in dystrophin/dysferlin-double null mice. Consistent with a model that GRAF1 facilitates dysferlin-dependent membrane patching, we found that GRAF1 associates with and regulates plasma membrane deposition of dysferlin.

**Conclusions:**

Overall, our work indicates that GRAF1 facilitates dysferlin-dependent membrane repair following acute muscle injury. These findings indicate that GRAF1 might play a role in the phenotypic variation and pathological progression of cardiac and skeletal muscle degeneration in muscular dystrophy patients.

**Electronic supplementary material:**

The online version of this article (doi:10.1186/s13395-015-0054-6) contains supplementary material, which is available to authorized users.

## Background

The plasma membrane (PM) functions as a physical barrier which protects the intracellular environment from the extracellular milieu, and continuous maintenance of this barrier is essential to support the proper function and vitality of all cells. The PM of striated muscle cells, or sarcolemma, is particularly susceptible to rupture as it endures significant mechanical stress and strain during muscle contraction. As such, the muscle must possess the ability to maintain sarcolemmal integrity and function, and previous studies have demonstrated that this is achieved by both sarcolemmal stabilization and dynamic sarcolemmal membrane repair.

Sarcolemmal stabilization in striated muscle is mediated in large part by the dystrophin glycoprotein complex (DGC) which localizes to the inner surface of the PM. The DGC functions primarily as a strong mechanical link between the intracellular cytoskeleton and the extracellular matrix (ECM) to stabilize the sarcolemma and transmit force laterally during muscle lengthening and contraction [1]. Integral components of the DGC include the transmembrane protein, β-dystroglycan, and its associated protein dystrophin, which connects the sarcolemma to the contractile machinery [1, 2]. In patients, a loss or reduction of dystrophin is causal for Duchenne muscular dystrophy or the milder Becker muscular dystrophy, respectively [3]. Studies in animal models have revealed that dystrophin deficiency increases sarcolemmal fragility in the skeletal muscles and in the heart and results in progressive muscle weakness and degeneration [4].

While the mechanistic details of sarcolemmal injury repair remain incompletely understood, studies indicate that membrane resealing involves coordinated changes in sub-plasmalemmal actin dynamics and intracellular vesicle transport. The current model suggests that membrane wounding results in the production of reactive oxygen species and an influx of Ca^2+^ that promotes transient sub-plasmalemmal actin polymerization, the oligomerization of repair proteins like the annexins [5] that prevent wound expansion, and the aggregation of intracellular vesicles (lysosomes or secretory vesicles) to the sub-plasmalemmal space. Following localized actin dissolution, vesicles fuse near the PM lesion area [6, 7], and the wound is eventually cleared from the membrane by either clathrin-independent endocytosis or shedding of micro-particles [8].

The first protein shown to actively participate in both skeletal and cardiac sarcolemmal injury repair was the 273-kDa membrane anchored calcium-binding protein, dysferlin. Mutations in dysferlin are causal for limb girdle muscular dystrophy 2B, Miyoshi myopathy [9, 10], and diseases also associated with skeletal and cardiac muscle degeneration [11–13]. Dysferlin-null mice exhibit cardiomyopathies when aged [14] and significant systolic dysfunction when stressed by conditions known to promote cardiomyocyte membrane disruption including exercise, isoproterenol (ISO) infusion, or genetic depletion of dystrophin [11, 15]. Importantly, in vitro experiments demonstrated that skeletal and cardiac muscle fibers lacking dysferlin exhibited defects in Ca^2+^-dependent membrane resealing following mechanical or laser-induced membrane rupture [16, 17]. Dysferlin is anchored to both intracellular vesicles and PMs, and its distribution between these compartments is tightly regulated by endocytic recycling [18]. In response to injury in normal muscle, dysferlin is rapidly recruited to PM-repair patches [17]. The finding that dysferlin-null muscle retained accumulation of vesicles near membrane damage sites indicates that dysferlin likely mediates the final step of docking and fusion of the “endomembrane patch” to reseal the membrane breach necessary for PM repair [19]; a function consistent with that of its closely related proteins, synaptotagmins, which are known to facilitate fusion of neurotransmitter-containing vesicles to the PM during exocytosis [20]. Notably, aggregation and reduced PM association of dysferlin are observed in several muscular dystrophies including caveolinopathies and sarcoglycanopathies suggesting that dysferlin mis-targeting could also play a role in the pathogenesis of these diseases [21–24]. However, further work is required to identify the precise molecular mechanisms that govern dysferlin targeting and vesicle recruitment to sites of membrane damage.

We previously showed that GRAF1 (guanosine triphosphatase (GTPase) regulator associated with FAK-1) is a Rho-specific GTPase activating protein that is expressed predominantly in striated muscle and that this protein mediates sarcolemmal recruitment of the fusogenic ferlin family members, myoferlin, and ferlin-1-like 5 (Fer1L5) to promote mammalian myoblast fusion and muscle growth [25–27]. Interestingly, depletion of GRAF1 from developing tadpoles led to a highly penetrable dystrophic phenotype and grossly impaired mobility, and ultrastructural analysis of the swimming muscles (i.e., somites) from these animals revealed numerous membrane tears [26]. Since tadpole somite muscle formation does not involve cell–cell fusion, these data indicated that GRAF1-depleted muscle fibers were either unable to withstand the mechanical strain or to repair strain-induced lesions that occurred during regular muscle usage. Herein, we show that GRAF1 is an essential component of the acute membrane repair response in both the skeletal and cardiac muscle. Overall, our mechanistic studies indicate that GRAF1 facilitates myoferlin/Fer1L5 dependent membrane fusion during muscle formation and dysferlin-dependent membrane repair following acute muscle injury.

## Methods

### Animals

Graf1 gene trap mice were generated and obtained from the Texas A&M Institute for Genomic Medicine (College Station, TX) and were described previously [27]. Mdx/C57/B10 mice were obtained from the Jackson Laboratory (Bar Harbor, ME). Experimental mice were generated by breeding the female mdx mice with the male Graf1 gene trap (GRAF1^Gt/Gt^) mice through two generations. The genetic background of all the experimental mice is a mixture of C57/B10 and C57BL/6J. F2 pups were genotyped for the Graf1 allele and the mdx allele as described previously [27, 28]. Echocardiographic analysis was performed in age- and littermate-matched female offspring. All other analyses were performed in age- and littermate-matched male offspring. The animals were treated in accordance with the approved protocol of the University of North Carolina (Chapel Hill, NC) Institutional Animal Care and Use Committee, which is in compliance with the standards outlined in the guide for the Care and Use of Laboratory Animals.

### Echocardiography

Left ventricular function was assessed by 2D echocardiography in conscious 7-month-old female mice using the Visualsonic Ultrasound System (Vevo 660) with a 30-MHz high-frequency transducer as described previously [29]. Echocardiographic measurements from three consecutive cycles were averaged using Visual Sonics software.

### Grip force measurements

Muscle forelimb grip strength was analyzed using an automated strain gauge as described previously [30].

### In vivo muscle injury models

To induce cardiac injury, Graf1^Wt/Wt^ (wild-type) and Graf1^Gt/Gt^ (GRAF1-depleted) mice were subject to intra-cardiac injections of 50 μL of 5 μM cardiotoxin (CTX) (*Naja nigricollis*, Calbiochem). After 24 h, the mice were subject to intraperitoneal injection of phosphate-buffered saline (PBS) containing 5 % Evans blue dye (EBD), and the hearts were harvested 24 h later. Alternatively, the mice were injected with EBD and subsequent injections of ISO (5 mg/kg) at 0, 16 and 23 h time points, and the hearts were harvested 1 h following the final injection. For both injury models, the blood was collected at time of tissue harvest and serum isolated to assess troponin T levels according to the manufacturer’s instructions (Life Diagnostics). To induce skeletal muscle injury, 50 μL of 20 μM CTX was injected into the quadriceps of 12-month-old male mice and the muscle was harvested 7 days later.

### Primary cell isolation, cell culture, and siRNA treatment

Primary and C2C12 mouse skeletal myoblasts were isolated and cultured as described previously [27]. Primary adult rat cardiomyocytes were isolated by the Langendorff method as described previously [31]. GRAF1 was depleted from cultured myocytes using short interfering RNA (siRNA) duplex oligoribonucleotides obtained from Invitrogen with the following sequences: graf1a sense 5′-GCAGCUGUUGGCCUAUAAU(dT)(dT)-3′ and anti-sense 5′-AUUAUAGGCCAACAGCUGC-3′, and graf1b sense 5′-AAGUGGACCUGGUUCGGCAACAUUU-3′ and anti-sense 5′-AAAUGUUGCCGAACCAGGUCCACUU-3′. The myocytes were transfected with 50 nM of Graf1 siRNA (25 nM of both graf1a and graf1b) or a GFP-specific siRNA as a non-target control using DharmaFECT reagent 1 according to the manufacturer’s instructions (Thermo Scientific). After 24 h, media was exchanged and cells were fixed.

### In vitro injury repair assays

For laser repair assay, Graf1^Wt/Wt^ and Graf1^Gt/Gt^ primary skeletal myoblasts were differentiated for 72 h before addition of FM 1-43 (Invitrogen), a membrane-impermeable dye, for 5 min prior to laser injury. Healthy, intact myotubes were targeted with a 10-s laser pulse, mode-locked on a 1 μm (l) × 0.5 μm (w) × 1 μm (d) region of the PM. Time-lapse images were acquired prior to and for up to 5 min following injury. The fluorescent intensity within (and remote to) the damaged site was quantified using Zeiss LSM 710 imaging software. Furthermore, to investigate GRAF1 redistribution to the disrupted PMs, the differentiated C2C12 myoblasts were mechanically injured with a scalpel blade or a microinjection needle (Eppendorf) 2 min prior to fixation and immunohistochemical analysis. For saponin repair assay, siRNA-treated primary cardiomyocytes were treated with FM 1–43 and permeabilized with 0.01 % saponin, or left untreated as a control, for 1 min prior to fixation.

### Immunohistochemistry and immunocytochemistry

The harvested mouse hearts and diaphragm muscles were immediately fixed in 4 % paraformaldehyde and processed for paraffin embedding using standard techniques. Unfixed frozen canine cranial tibialis muscles, a generous gift from Dr. Joe Kornegay, were fixed and processed as above. Alternatively, the mouse tibialis anterior muscles were immediately embedded in Tissue-Tek O.C.T. compound (Sakura) and snap-frozen in 2-methylbutane cooled over dry ice. For immunohistochemistry, the tissues were cross-sectioned at 8 μm, post-fixed in 4 % paraformaldehyde (frozen sections), treated for antigen retrieval using 10 mmol/L citrate buffer (pH 6.0), and stained using standard techniques. For immunostaining of the cultured myocytes, the cells were fixed in 4 % paraformaldehyde and permeabilized using PBS containing 0.1 % Triton X-100 and 0.1 % sodium citrate (for cardiac myocytes) or PBS containing 0.4 % Triton A-100 (for skeletal myocytes). The tissues/cells were incubated with primary antibodies at 1:200 dilutions at 4 °C overnight. Commercial antibodies were purchased from Sigma (laminin, monoclonal γ-tubulin), Abcam (α-actinin), Lifespan Biosciences (annexin A1), Leica Microsystems (dysferlin), and Developmental Studies Hybridoma Back, University of Iowa (eMHC, troponin T). Derivation of the GRAF1 rabbit and hamster antibodies were described previously [27]. The tissues were then incubated with Alexa Fluor secondary antibodies (Invitrogen), Alexa Fluor phalloidin (Invitrogen), Alexa Fluor wheat germ agglutinin (Invitrogen), and DAPI at 1:500 in PBS for 1 h, washed, and mounted. Fluorescent images were acquired using a Zeiss LSM 710 confocal laser-scanning microscope. ImageJ software was used to quantify the myofiber cross-sectional area and the in vivo fusion index as previously described [27].

### Histological analysis

The tissue sections processed as above were subjected to hematoxylin and eosin (H&E) staining using standard techniques, Masson trichrome (MTC) staining (Sigma) or Picrosirius red staining (Polysciences) according to the manufacturer’s instructions, and visualized using bright field microscopy. To assess cardiac fibrosis, the cross-sections of MTC-stained hearts were scored as follows: 1 = 0–50 % of vessels exhibited peri-vascular fibrosis with minimal/no interstitial fibrosis, 2 = 50–80 % of vessels exhibited peri-vascular fibrosis with minimal/no interstitial fibrosis, 3 = 50–80 % of vessels exhibited peri-vascular fibrosis with intermediate interstitial fibrosis and presence of scar, and 4 = 80–100 % of vessels exhibited vascular fibrosis with significant interstitial fibrosis and scar. To quantify diaphragm thickness, cross-sectional widths along the length of a tissue section were measured and averages calculated using ImageJ software. To quantify diaphragm myofiber number, the diaphragm cross-sections were immunostained to demark myofiber boundaries and imaged using confocal microscopy. The total myofiber number from three representative images that spanned 650 um in length and the total cross-sectional width of the diaphragm were quantified using ImageJ, and these values were used to derive the total number of myofibers per diaphragm. To visualize diaphragm fibrosis, Sirius red-stained tissues were first imaged under linear polarized light using identical gain. ImageJ was then used to quantify the integrated density of the red and green signal from each image. Collagen composition is described as the average ratio of red to green signal density per area of tissue. Images were acquired using an Olympus BX61 microscope.

### Co-immunoprecipitation

For immunoprecipitation studies, isolated mouse quadriceps muscle was sonicated in modified radioimmune precipitation assay (RIPA) buffer (50 mM HEPES pH 7.2, 0.15 M NaCl, 2 mM EDTA, 0.1 % Nonidet P-40, 0.05 % sodium deoxycholate, 0.5 % Triton X-100 plus 1 mM sodium orthovanadate and 1× concentrations of both Halt Protease Inhibitor Cocktail (Thermo Scientific) and Halt Phosphatase Inhibitor Cocktail (Thermo Scientific) and cleared by centrifugation. One thousand microgram of cleared lysate was incubated with 10 μg of either an anti-GRAF1 antibody (polyclonal) overnight at 4 °C. The solution was then mixed with 75 μL of a 50 % slurry of Protein A Sepharose beads (Sigma) in tris-buffered saline (TBS) and rotated at 4 °C for 2 h. the beads were then quickly tapped down in a refrigerated centrifuge and rinsed three times with ice-cold RIPA + inhibitors and once with TBS before the beads were boiled in 50 μL of sample buffer. Eluates and a 2 % lysate input were resolved by SDS-PAGE, transferred to nitrocellulose membranes, and immunoblotted with an anti-dysferlin antibody and an anti-GRAF1 antibody (monoclonal) at 1:1000 dilutions using standard techniques.

### Electroporation, fiber preparation, and laser damage assay

Flexor digitorum brevis fibers were transfected with an N-terminally tagged mCherryGRAF1 complementary DNA (cDNA) using methodology similar to the in vivo electroporation methods previously described [32]. Briefly, the footpads of wild-type (WT)^129^ mice were injected with 10 μl of hyaluronidase (8 units). Two hours post injection, mCherryGRAF1 cDNA (2 μg/μl) was injected into the footpad and the muscle was stimulated. The muscle was allowed to recover for 7 days, and then fibers were isolated. The flexor digitorum brevis muscle was dissected and placed in DMEM containing BSA plus collagenase type 2 solution. Dissociated fibers were imaged on Matek confocal microscopy plates (P35G-1.5-14-C; Matek). Utilizing LAS AF Leica Imaging Software, membrane lesions were induced using FRAP Bleach point in the fluorescence recovery after photobleaching (FRAP) wizard protocol. The 405-nm laser was set at 80 % power for 3 s, and images were acquired on a Leica SP5 2 photon microscope. A single image was acquired before damage, upon laser damage, and every 2 s after damage, and then one image every 10 s.

### Statistical analyses

Student *t* test was applied for comparison of means. Analysis of variance (ANOVA) was applied for comparison between groups. Data are represented as mean ± standard error of mean (sem), and *p* values <0.05 were considered statistically significant.

## Results and discussion

### GRAF1 is dynamically recruited to damaged PMs

**Fig. 1:**
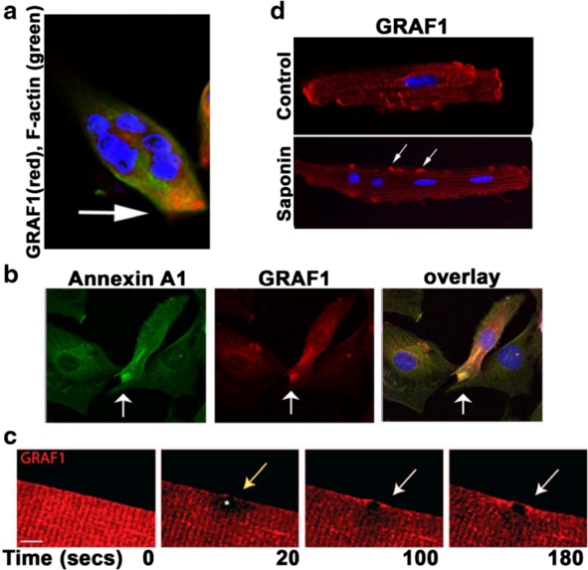
GRAF1 accumulates at PM injury sites. **a** A 72-h differentiated C2C12 myoblast cut with a scalpel blade was fixed 2 min post-injury and stained for GRAF1 (*red*). Note accumulation of GRAF1 at the site of injury. F-actin and nuclei were counterstained with phalloidin (*green*) and DAPI (*blue*), respectively. **b** GRAF1 (*red*) co-localizes with the membrane fusion/repair protein, annexin A1 (*green*) at site of microinjection needle-mediated PM puncture in cultured myoblasts. Cells were fixed and stained 2 min after injury. **c** mCherry-GRAF1 labeled myofibers were laser damaged, and GRAF1 localization was monitored over time. Note that visible accumulation of GRAF1 at the site of injury (denoted by *asterisk*) by 100 s post-injury. **d** Immunohistochemical analysis of GRAF1 expression (*red*) in control and saponin-treated cultured adult rat ventricular cardiomyocytes. Note that the translocation of GRAF1 to the PM of saponin-treated cells (*white arrows*). Nuclei are counterstained with DAPI (blue)

We previously showed that GRAF1 was dynamically recruited to the tips of fusing myoblasts wherein it promoted membrane merging by facilitating ferlin-dependent capturing of intracellular vesicles [27]. Because acute membrane repair responses involve similar vesicle trafficking complexes/events and because our prior studies in GRAF1-deficient tadpoles revealed a putative role for GRAF1 in maintaining muscle membrane integrity [26], we postulated that GRAF1 might also play an important role in facilitating PM repair. To test this hypothesis, we first sought to determine if GRAF1 was recruited to sites of sarcolemmal injury. In support of a role for GRAF1 in mediating acute injury repair, we found that mechanical rupture of C2C12 myotubes resulted in the rapid (within 2 min) recruitment of endogenous GRAF1 from peri-nuclear compartments to the site of membrane lesions where it co-localized with the membrane repair protein, annexin A1 (Fig. 1a, b). Co-staining with phalloidin indicated that the sites of sarcolemmal injury were nearly completely devoid of actin-based structures, consistent with our previous finding that when targeted to the membrane, GRAF1 promoted marked clearing of cortical F-actin [26]. In mature skeletal and cardiac muscle fibers, endogenous GRAF1 was predominantly cytoplasmic and was distributed along Z-bands (Fig. 1c, d). To determine if damage also promoted GRAF1 sarcolemmal recruitment in mature muscle fibers, we electroporated mCherryGRAF1 into native adult skeletal myofibers and induced damage by laser wounding using previously established methods [33]. Live cell imaging revealed that GRAF1 rapidly translocated from Z-bands to the sarcolemmal injury site in these cells (Fig. 1c). For cardiomyocytes which are too small for laser injury, we utilized saponin treatment to induce injury [34]. Similarly, we found that saponin treatment induced translocation of endogenous GRAF1 from Z-bands to distinct PM sites on adult cardiomyocytes (Fig. 1d). Collectively, these findings are reminiscent of studies in which sarcolemmal injury induced the rapid accumulation of dysferlin that other proteins implicated in repair including the following: annexins A1, A2, and A6 [33, 35], caveolin-3 [21], AHNAK [36], and mitsugumin 53 (MG53) [22]. As each of these proteins has been implicated in mediating myoblast membrane repair [5, 22, 33, 37–41], we reasoned that GRAF1 might be an additional essential component of this membrane repair “kit”.

**Fig. 2:**
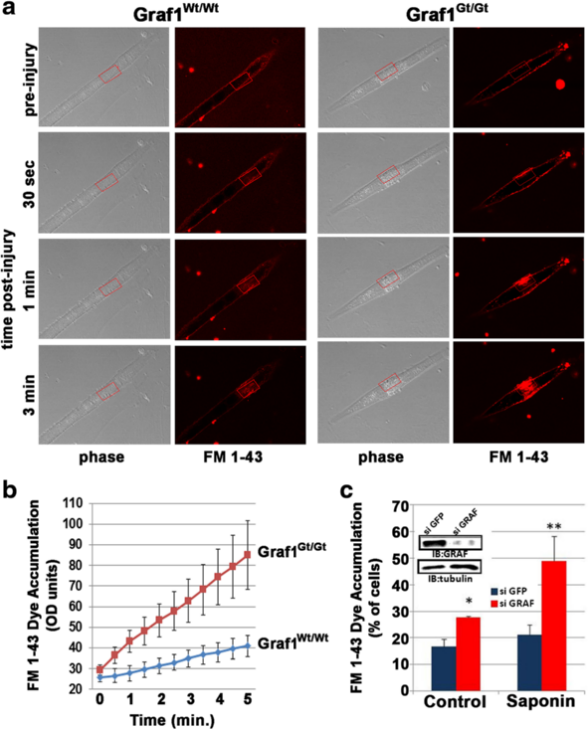
GRAF1 regulates skeletal and cardiac muscle membrane repair. **a** Graf1^Wt/Wt^ and Graf1^Gt/Gt^ primary myotubes were treated with FM 1-43 membrane dye (*red*) for 5 min and imaged prior to confocal-directed laser injury (1 μM × 0.5 μM within *red square*; *top panels*) and for 3 min post-injury. Note the pronounced FM 1-43 accumulation in Graf1^Gt/Gt^ cells. **b** Quantification of dynamic FM 1-43 dye accumulation following injury (*n* = 23–28 cells per genotype; *N* = 5). Data are represented as mean ± sem **c** Quantification of FM 1-43 dye-positive cardiomyocytes treated with or without saponin following transfection with control (GFP) or Graf1-specific siRNAs. Note the significant increase in dye accumulation in GRAF1-depleted cells. *Western blot inset* demonstrates efficient Graf1 knockdown. γ-tubulin is shown as the loading control

### GRAF1-depleted muscle cells have reduced membrane healing abilities

We next used an in vitro laser injury model to test whether GRAF1 was necessary for efficient muscle membrane repair. Primary myoblasts isolated from a previously described GRAF1-deficient mouse line (GRAF1^gt/gt^) and a littermate control line (GRAF1^wt/wt^) were subjected to differentiation media for 48 h, and laser injury was performed on bi-nucleated myotubes that had been pre-incubated with membrane-impermeable FM 1-43 dye (Fig. 2a). Time-lapse images revealed efficient PM resealing following laser injury of GRAF1^wt/wt^ myotubes as demonstrated by a transient increase in FM 1-43 fluorescence within the wounded region (Fig. 2a, b). In stark contrast, when subjected to an identical laser burst, myocytes isolated from GRAF^gt/gt^ mice exhibited a pronounced and widespread increase in FM 1-43 fluorescence, indicative of failed initial attempts at membrane resealing [42]. Pronounced resealing defects were also observed in GRAF1-depleted cardiomyocytes both prior to and following saponin treatment as assessed by dye accumulation (Fig. 2c). Collectively, these studies support the postulate that GRAF1 plays a critical role in resealing membranes damaged by mechanical stress.

### GRAF1 depletion compromises sarcolemmal integrity in vivo

As noted above, we recently developed GRAF1 hypomorphic mice using an available GRAF1 gene trap ES line (Texas Genome Institute of Medicine) and Western blot analysis confirmed that the gene trap led to a near complete depletion of GRAF1 in the heart and various skeletal muscles [27]. Unlike in GRAF1-depleted tadpoles [26], there was no indication of premature lethality in GRAF1-depleted mice and we showed that this may be due, at least in part, to functional compensation by the closely related family member GRAF2 [27]. Nonetheless, some hallmarks of muscular dystrophy were apparent in GRAF1^gt/gt^ skeletal muscles including centro-nucleated atrophic fibers and myofiber branching [27], and our recent studies show that 6-month-old GRAF1^gt/gt^ hearts exhibited significantly higher levels of fibrosis, indicative of cardiac muscle degeneration (see Fig. 5). Moreover, ultrastructural analysis revealed that GRAF1-depleted muscle exhibited elongated T-tubules and vacuole-T-tubule fusion (Additional file 1: Figure S1). These defects are hallmarks of dysferlinopathies [43, 44] and could contribute to dystrophic pathological progression in this model because T-tubules serve as a membrane source for rapid sarcolemmal repair [45, 46]. Thus, GRAF1 depletion phenocopies dysferlin deficiency as (1) depletion of either protein in cultured skeletal and cardiac cells leads to delayed myoblast membrane resealing [17] and (2) depletion of either gene in mice leads to a modest cardiac and skeletal muscle dystrophy [11, 14, 15, 17, 47].

**Fig. 3:**
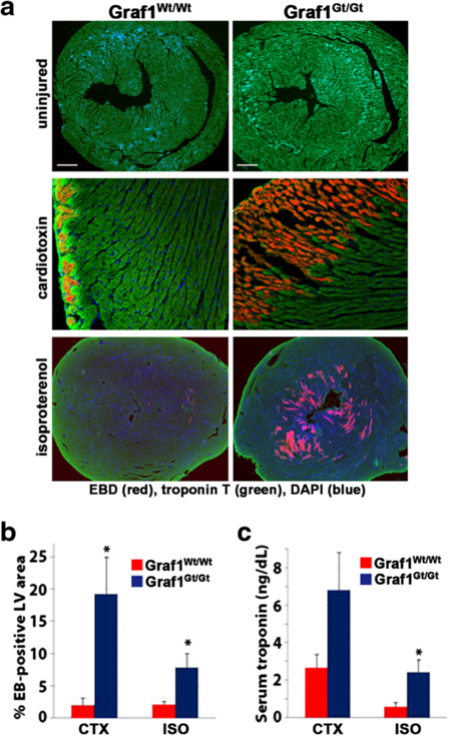
GRAF1 is required for optimal cardiomyocyte membrane repair in vivo. **a** Representative images of Evans blue dye (*EBD*; *red*) uptake in the hearts from Graf1^Wt/Wt^ and Graf1^Gt/Gt^ adult mice prior to (*top panels*) and 24 h after subjection to intra-cardiac injection of cardiotoxin (CTX) (*middle panels*) or i.p. injection of isoproterenol (ISO) (*bottom panels*). Cardiomyocytes and nuclei are counterstained with troponin T antibody (*green*) and DAPI (*blue*), respectively. **b** Graphical representation of the area of EBD uptake in the left ventricle (LV) cross-section of injured hearts. **c** Quantification of serum troponin levels in injured mice. Note the significant increases in markers of cardiomyocyte damage in GRAF^gt/gt^ mice. (*N* = 6–7 mice per genotype). Data are represented as mean ± sem. Scale bars = 20 μm

To further explore the role for GRAF1 in dysferlin-dependent maintenance of cardiac membrane integrity, we challenged young 2–3-month-old GRAF1^gt/gt^ mice with agents including CTX or the contractile agonist ISO that have been previously shown to induce marked sarcolemmal injury in dysferlin-null mice [11, 47]. In brief, the GRAF1^gt/gt^ mice were subjected to cardiotoxin injection (50 μl of 5 μM) into the left ventricular wall or to 3 × 5 mg/kg intraperitoneal (i.p.) injections of ISO over a 24-h period. EBD was injected i.p. immediately following injury, and sarcolemmal damage was evaluated in these models by assessing its cellular accumulation. As previously reported, EBD binds avidly to serum albumin and as such dye can only enter cells that have compromised PMs. As shown in Fig. 3a, EBD uptake was not apparent in non-treated GRAF1^wt/wt^ or GRAF1^gt/gt^ hearts and CTX injection into control hearts led to modest EBD accumulation that was restricted to the sub-epicardial zone (red, EBD; green, cardiac troponin T). In stark contrast, large clusters of EBD-positive cardiomyocytes were apparent in the myocardial wall adjacent to the CTX injection site in the GRAF^gt/gt^ hearts. Likewise, ISO treatment of the GRAF1^gt/gt^ mice led to significantly increased accumulation of myocardial EBD in comparison to similarly treated GRAF1^wt/wt^ mice (Fig. 3a, b). Concomitantly, serum troponin levels were significantly higher in ISO-treated GRAF^gt/gt^ mice relative to similarly treated GRAF1^wt/wt^ littermate controls (Fig. 3c). Collectively, these data indicate that like dysferlin, GRAF1 plays a critical role in maintaining cardiomyocyte PM integrity and in facilitating intrinsic cardiomyocyte membrane repair processes that can occur during common pathological insults.

### Dystrophin deficiency exacerbates cardiac and skeletal muscle pathologies in GRAF1-depleted mice

**Fig. 4:**
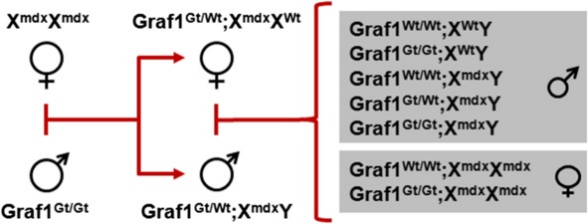
Breeding scheme for the generation of dystrophin/GRAF1-deficient mice. Genotypes of experimental mice are denoted in *gray boxes*. Graf1^Wt/Wt^; X^Wt^X^Wt^ or X^Wt^Y = wild-type; Graf1^Gt/Gt^; X^Wt^X^Wt^ or X^Wt^Y = Graf1-deficient; Graf1^Wt/Wt^; X^mdx^X^mdx^or X^mdx^/Y = dystrophin-deficient; Graf1^Gt/Wt^;X^mdx^X^mdx^ or X^mdx^/Y = Graf1 haploinsufficient/dystrophin-deficient; Graf1^Gt/Gt^; X^mdx^X^mdx^or X^mdx^/Y = Graf1/dystrophin double-deficient

**Fig. 5:**
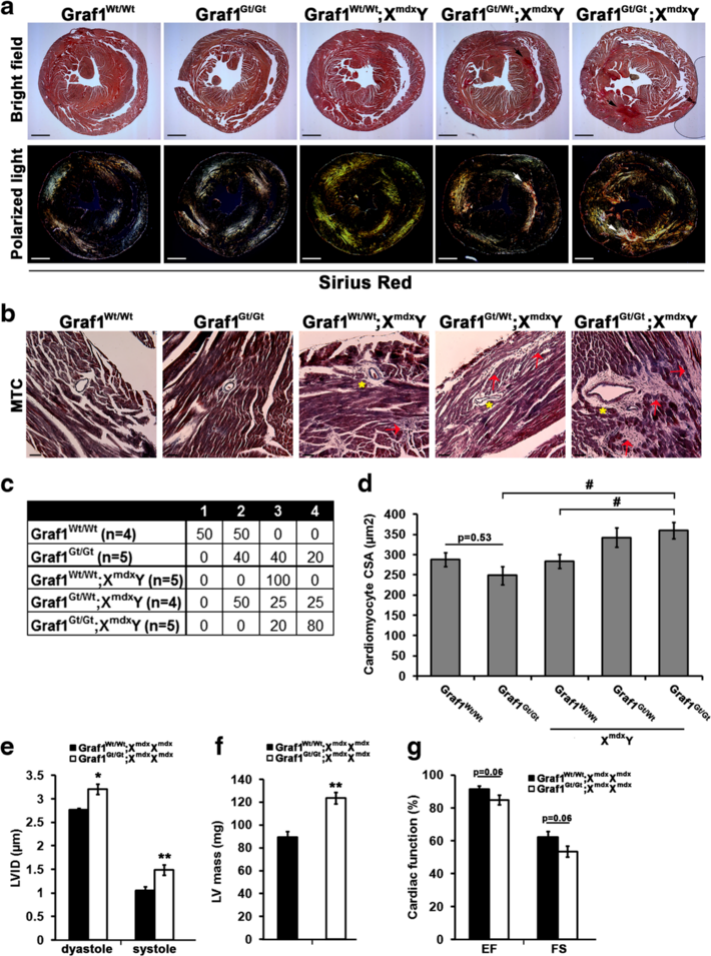
GRAF1 depletion exacerbates cardiac fibrosis and reduces cardiac output in *mdx* mice. **a** Ventricle cross-sections from 6-month-old male mice with indicated genotypes stained with Sirius red and visualized using bright field microscopy (*upper panels*) or under polarized light (*bottom panels*). Note the fibrotic patches in double-depleted hearts (*arrowheads*). Scale bars = 1.0 mm. **b** Ventricle sections stained with Masson’s trichrome (MTC) reveals fibrosis as well as peri-vascular (*asterisk*) and interstitial inflammation (*red arrowheads*) in GRAF1/dystrophin-deficient hearts. Scale bars = 50 μm. **c** Percentage of mice with cardiac fibrosis at 6 months of age. Cross-sections of MTC-stained hearts were quantitated as follows: 1: 0–50 % peri-vascular fibrosis; minimal interstitial fibrosis; no scarring. 2: 50–80 % peri-vascular fibrosis; minimal interstitial fibrosis; no scarring. 3: 50–80 % peri-vascular fibrosis; intermediate interstitial fibrosis; some scarring. 4: 80–100 % peri-vascular fibrosis; significant interstitial fibrosis; significant scarring. **d** Average cardiomyocyte cross-sectional area (CSA) from hearts of 6-month-old mice. **e**–**g** Conscious echocardiographic analysis of left ventricular internal diameter (LVID) LV mass, ejection fraction (EF), and fractional shortening (FS), respectively, in 7-month-old female mice. (*p < 0.005, **p < 0.01, ^#^ *p* < 0.05; *N* = 4–5 mice per genotype). Data are represented as mean ± sem. Body weights for female mice were as follows and were not significantly different between groups Graf1^Wt/Wt^; X^mdx^X^mdx^ (30.4 ± 3.4 g); Graf1^Gt/Wt^;X^mdx^X^mdx^ (31.0 ± 3.4 g); Graf1^Gt/Gt^;X^mdx^X^mdx^ (31.1 ± 3.6)

Studies from the Campbell lab have revealed that DGC-mediated membrane stability and dysferlin-mediated membrane repair are both necessary for the homeostatic maintenance of striated muscle membrane integrity. Indeed, they showed that combined deficiency of dysferlin and dystrophin led to more severe muscle pathology resulting from increased susceptibility to muscle membrane rupture and to defective membrane repair [11, 48]. Therefore, we postulated that if GRAF1 was indeed an essential mediator of dysferlin-dependent membrane repair, then co-depletion of dystrophin would unmask this function and exacerbate cardiac and skeletal defects in GRAF1^gt/gt^ mice. To test this hypothesis, dystrophin/GRAF1 double-deficient offspring were generated using the breeding scheme depicted in Fig. 4 (refer to figure legend for description of mouse nomenclature). As shown in Fig. 5a, both GRAF1- and dystrophin-deficient (*mdx*) mice present with modest cardiac pathology at 6–7 months of age as assessed by the presence of interstitial “myocyte replacement” fibrosis (i.e., collagen deposition as visualized by Sirius red staining under polarized light) when compared to GRAF1^wt/wt^ (WT) controls. However, consistent with the postulate that GRAF1 deficiency would render myocytes more sensitive to membrane instability, the hearts from GRAF1/dystrophin double-deficient (GRAF1^gt/gt^;X^mdx^Y) mice exhibited severe pathology, including widespread fibrosis, focal necrosis and inflammation, and hypertrophic cardiomyocyte growth (Fig. 5a–d). Accordingly, we found that double depletion of dystrophin and GRAF1 led to reduced cardiac performance as assessed by conscious echocardiography. We used female mice for the functional studies, because significant differences in body weights were observed in the male but not female mice (see below for further discussion and Additional file 1: Figure S2). As shown in Fig. 5e–g, despite no significant difference in body size, the hearts from the compound GRAF1^gt/gt^;X^mdx/mdx^ mice exhibited a significant increase in left ventricle (LV) mass and LV chamber diameter accompanied by decreased ejection fraction and fractional shortening, indicative of cardiac dysfunction. Together, these findings support the possibility that compound dystrophin/GRAF1 deficiency (like compound dystrophin/dysferlin deficiency) leads to progressive myocyte necrosis due to cumulative damage coupled with insufficient membrane repair responses.

**Fig. 6:**
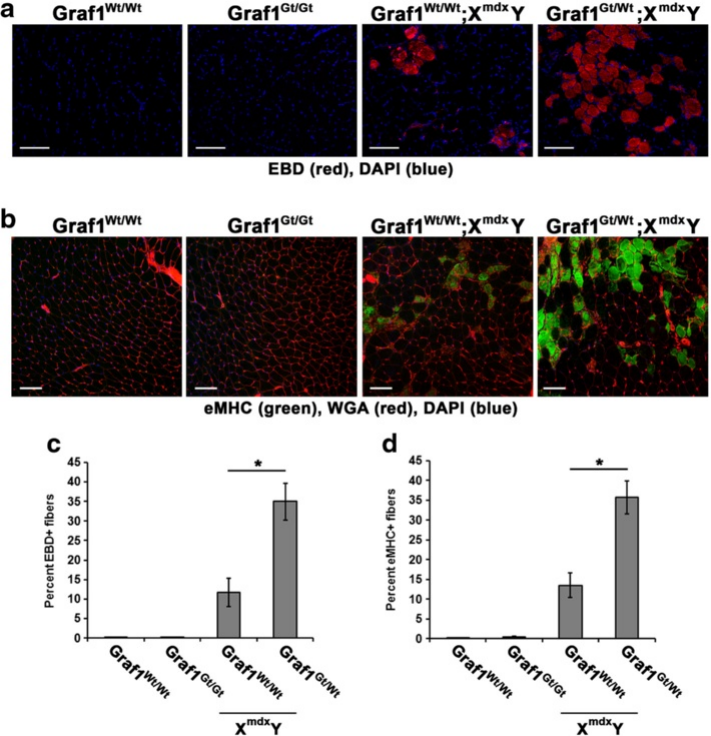
GRAF1 partial depletion increases EBD accumulation and myofiber regeneration in young *mdx* muscle. **a** Representative images of EBD (*red*) accumulation in tibialis anterior muscle of 3-week-old littermates with indicated genotypes. The mice were injected with EBD 24 h prior to tissue harvest. Nuclei are counterstained with DAPI (blue). **b** Three-week-old tibialis anterior muscle immunostained with eMHC (*green*) to demarcate regenerative fibers. Wheat germ agglutinin (WGA; *red*) demarcates myofiber boundaries. Nuclei are counterstained with DAPI (*blue*). **c**, **d** Percentage of EBD- and eMHC-stained tibialis anterior myofibers, respectively, (*p < 0.005; *N* = 3 per genotype). Data are represented as mean ± sem. Scale bars = 100 μm

To more directly test the postulate that GRAF1 regulates a dysferlin-associated membrane repair function in vivo, we further explored skeletal muscle sarcolemmal integrity in GRAF1-deficient mdx mice. It is well documented that *mdx* mice undergo a period of acute skeletal myofiber necrosis and degeneration/regeneration that begins around 3–4 weeks of age. To assess membrane integrity at this stage of development, we injected 3-week-old pups with EBD and quantified dye uptake 24 h later. Due to limited offspring of double-deficient mice, we evaluated muscles from the *mdx* mice that were Wt or heterozygous at the GRAF1 locus. While punctate areas of EBD-positive fibers (indicative of focal necrosis) were detected in the *mdx* tibialis anterior muscle, widespread EBD accumulation was observed in the muscles isolated from mdx mice with partial GRAF1 depletion (GRAF1^gt/wt^;X^mdx^Y mice Fig. 6a, c). The finding that GRAF1^gt/gt^ muscles did not exhibit detectible levels of EBD-positive muscle fibers at this time point indicates that depletion of GRAF1 does not destabilize DGCs at the sarcolemma. Skeletal muscle membrane disruptions not only permit EBD uptake but also initiate a regenerative response which can be assessed by embryonic myosin heavy chain (eMHC) expression in nascent fibers. Consistent with our EBD uptake assay, a significant increase in the number of eMHC-positive fibers was found in GRAF1^gt/wt^X^mdx^Y muscles relative to the other genotypes tested (Fig. 6b, d). Collectively, these data indicate that the degeneration/regeneration phenotype observed in neonatal *mdx* mice was exacerbated by partial depletion of GRAF1.

We next analyzed the consequence of GRAF1 and dystrophin deficiency in young adult mice. As mentioned above, total body weights of 6-month-old male mice increased significantly in GRAF1^gt/gt^;X^mdx^Y mice relative to age-matched and littermate control mice and this effect was primarily due to increased muscle mass (Additional file 1: Figure S2). These data are reminiscent of findings in DMD patients who frequently exhibit a transient increase in body weight and muscle mass at young ages [9, 10, 49, 50]. Histological analysis of the tibialis anterior muscles revealed that the increase in mass observed in the GRAF1^gt/gt^;X^mdx^Y muscle was due to myofiber expansion not myofiber hypertrophy (Additional file 1: Figure S3). Indeed, the GRAF1^gt/gt^;X^mdx^Y tibialis anterior muscles contained significantly more myofibers per unit area and, as demonstrated in the frequency histogram, this was accompanied by a higher percentage of small regenerating myofibers. Notably, at this time point the *mdx* and GRAF1^gt/gt^;X^mdx^Y muscles contained a comparably high percentage of regenerating fibers (nearly 90 % as demarcated by centrally localized nuclear foci) indicating that the expansion of fibers in the GRAF1^gt/gt^;X^mdx^Y muscles was most likely due to continued rounds of degeneration/regeneration. Studies in dysferlin-null mice have also reported that injury recovery occurs by a marked increase in myogenesis [51], though the proliferative effects in double-depleted GRAF1^gt/gt^;X^mdx^Y mice appear to be more profound than previously reported in the mdx/dysferlin-depleted mice [48]. This difference may be due, in part, to the limited capacity for nascent GRAF1^gt/gt^ myoblasts to fuse, which when coupled with cumulative defects in membrane repair could lead to the increased production of small fibers. However, despite fiber size variability, the GRAF1^gt/gt^;X^mdx^Y tibialis anterior muscles did not exhibit any overt pathology or functional deficit (data not shown) at this time point.

**Fig. 7:**
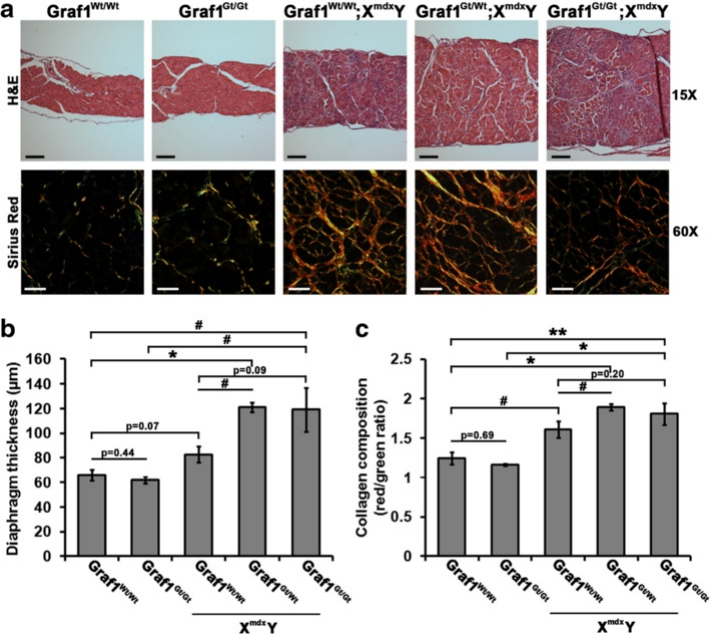
Histopathological analysis of diaphragm muscles in GRAF1/dystrophin-deficient mice. **a** Diaphragm cross-sections from 6-month-old male mice with indicated genotypes stained with hematoxylin and eosin (H&E) (*top panels*) or Sirius red and imaged under polarized light to visualize fibrosis (*bottom panels*). **b** Quantification of average diaphragm thickness from 6-month-old male mice. **c** Graphical representations of collagen composition of diaphragms from 6-month-old male mice (refer to methods for quantification details; *p < 5 × 10^−4^; **p < 0.01; ^#^ *p* < 0.05; *N =* 5 mice per genotype). Data are represented as mean ± sem. *Black scale bars* = 25 μm; *white scale bars* = 100 μm

Since the diaphragm muscle is known to be more susceptible to defects in membrane injury repair, we reasoned that these muscles might exhibit a more severe dystrophic phenotype in the GRAF1/dystrophin-deficient mice. Indeed, while we found no difference in the diaphragm diameter between Wt and GRAF^gt/gt^ mice, the diaphragms of *mdx* mice were slightly larger—consistent with ongoing tissue damage and repair—and further increases were observed in the diaphragms isolated from GRAF1^gt/wt^;X^mdx^Y and GRAF1^gt/gt^;X^mdx^Y mice (Fig. 7a top, b; note the similar effect of GRAF1 haplo- and homo-deficiency). Importantly, the increase in diameter in the GRAF1-deficient *mdx* diaphragms was accompanied by a profound increase in ECM deposition as assessed by Sirius red staining which revealed enhanced red birefringence indicative of increased deposition of type I collagen and other highly ordered collagens (Fig. 7a bottom, c) as well as an increase number of myofibers (*p* = 0.06, GRAF1^gt/gt^;X^mdx^Y relative to *mdx*; Additional file 1: Figure S4a). Interestingly, immunostaining for eMHC, to demarcate regenerating myofibers, revealed that while *mdx* mice exhibited numerous eMHC-positive fibers at this 6-month time point, the GRAF1^gt/wt^;X^mdx^Y and GRAF1^gt/gt^;X^mdx^Y diaphragms exhibited minimal eMHC staining (Additional file 1: Figure S4b, green) and a reduction in central nucleation (*p* = 0.06, GRAF1^gt/gt^;X^mdx^Y relative to *mdx*; Additional file 1: Figure S4c). These finding are consistent with a progressive exhaustion of regenerative potential in these diaphragm muscles resulting from reduced DGC-dependent membrane stability coupled with reduced GRAF1-dependent repair.

### GRAF1 co-associates with dysferlin and regulates its PM recruitment

**Fig. 8:**
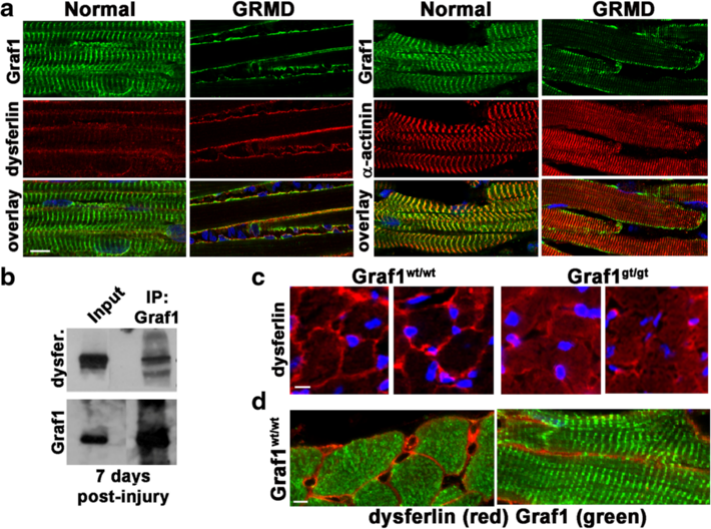
GRAF1 associates with dysferlin in injured/dystrophic muscles and promotes its recruitment to the PM. **a** Immunohistochemical analysis of GRAF1 expression (*green*) in normal and dystrophic (golden retriever muscular dystrophy; GRMD) muscle. Co-staining with dysferlin (*red*, *left*) and α-actinin (*red*, *right*) demonstrates localization of GRAF1 in striated pattern aligned with Z-disks in normal muscle compared to localization to the plasmalemma in GRMD muscle (note intramuscular inflammatory infiltrates indicative of muscle injury as visualized by DAPI staining). **b** Anti-GRAF1 rabbit polyclonal antibody [52] immunoprecipitation (IP) from adult quadriceps muscle 7 days following CTX-induced injury. Blots were probed with an anti-dysferlin antibody or hamster anti-GRAF1 antibody. *Input* contains 2 % of cellular lysate used for IP. **c** Immunohistochemical analysis of dysferlin (*red*) in Graf1^Wt/Wt^ and Graf1^Gt/Gt^ adult mouse hearts indicates dysferlin mis-localization in the absence of GRAF1. Nuclei are counterstained with DAPI (blue). **d** Immunohistochemical analysis of dysferlin (*red*) and GRAF1 (*green*) reveals plasmalemmal and sarcomeric localization respectively in Wt hearts. Scale bars = 20 μm

Dysferlin is anchored to both intracellular vesicles and PMs, and its distribution between these compartments is tightly regulated by endocytic recycling. In response to injury in normal muscle, dysferlin is rapidly recruited to PM-repair patches [17] though the underlying mechanisms remain unclear. Since GRAF1 depletion phenocopies dysferlin deficiency and since our previous studies revealed that GRAF1 promoted cell–cell fusion by facilitating the endocytic vesicle to PM trafficking of the dysferlin family members, myoferlin and Fer1L5, we reasoned that GRAF1 might facilitate rapid membrane repair, at least in part, by regulating recycling-dependent accumulation of dysferlin to PM injury sites. We first explored dysferlin and GRAF1 localization in neonatal muscles isolated from 9-day-old control or dystroglycan-deficient golden retriever muscular dystrophy (GRMD) that model Duchene’s muscular dystrophy, since these animals (unlike the *mdx* mice) exhibit significant and widespread muscle pathology at a young age [52] when GRAF1 is most robustly expressed [25–27]. As shown in Fig. 8a, GRAF1 was almost exclusively localized in a striated pattern aligned with Z-disks in normal muscle while dysferlin was predominantly localized to the sarcolemma. In contrast, GRAF1 exhibited remarkable plasmalemmal accumulation and co-localization with dysferlin in GRMD muscle. We observed a similar plasmalemmal accumulation of GRAF1 in CTX-injured mouse skeletal muscles (data not shown) and accordingly, co-IP revealed a strong interaction between GRAF1 and dysferlin in these fibers (Fig. 8b). Moreover, while dysferlin was predominantly localized to the PM of cardiac muscle in Wt mice (and GRAF1 to sarcomeric structures), dysferlin exhibited diffuse cytoplasmic staining in GRAF1^gt/gt^ mouse hearts, indicating that GRAF1 is important for the trafficking of dysferlin to the PM (Fig. 8c, d). This finding is consistent with the model first described by us [27] and recently confirmed by others [53] that GRAF1 forms a complex with the C-terminal Eps15 homology domain (EHD) protein EHD1, a protein involved in membrane vesiculation and the transport of myoferlin and Fer1L5 to the membrane [54]. Specifically, GRAF1 appears to be important in promoting the exit of such critical cargo molecules from the endocytic recycling compartment to the PM [53]. This process is likely governed by the membrane curvature inducing abilities of the Bin–amphiphysin–Rvs (BAR) and pleckstrin homology (PH) domains of GRAF1 which facilitate the budding of small and motile recycling endosomes from the static tubular endocytic compartment. Because GRAF1 also functions to accelerate actin de-polymerization, we favor a model in which GRAF1 may associate with vesicles through its BAR domain and that vesicle-associated GRAF1 facilitates clearing of sub-plasmalemmal actin to aid in ferlin-dependent vesicle capture. The GRAF1-labeled vesicles might be specifically targeted to sites of membrane injury through its PH domain, because this domain has a high affinity for phosphatidyl serine, and phosphatidyl serine is known to be exposed on the inner leaflets of injured membranes and the outer leaflets of exocytic vesicles [37]. However, future studies will be necessary to determine the extent that dysferlin mis-localization is a direct consequence of GRAF1 depletion or is secondary to a general dystrophic phenotype as intracellular accumulation of dysferlin has also been observed in dystrophic muscles of patients with various clinical diagnoses [55, 56].

## Conclusions

Previous studies demonstrated that dysferlin-null skeletal myoblasts exhibited delayed membrane patching and a more severe injury following sarcolemmal damage [17]. Our findings indicate that GRAF1 deficient skeletal and cardiac muscle cells exhibit similar defects in acute membrane resealing following injury. Moreover, we show that dystrophin depletion exacerbated muscle damage in GRAF1-deficient mice and that mice with dystrophin/GRAF1 double deficiency phenocopied the severe muscle pathologies observed in dystrophin/dysferlin-double null mice [48]. Consistent with a model that GRAF1 facilitates dysferlin-dependent membrane patching, we found that GRAF1 associates with and regulates PM deposition of dysferlin. Interestingly, it was recently shown that null mutations in dysferlin that render myocytes incapable of rapid vesicle-mediated membrane repair also result in defects in vesicle-dependent exocytosis of chemotactic factors including MCP-1 that are necessary for efficient muscle regeneration [47]. Whether GRAF1, like dysferlin, also mediates the release of chemotactic molecules will be an important focus of future studies. Importantly, variations of GRAF1 expression are common in the human population and are associated with many diseases ranging from X-linked alpha-thalassemia mental retardation syndrome [57] to adenocarcinomas and myelodysplastic syndrome [58–61]. Our findings indicate that the extent to which GRAF1 variations modify the onset and/or severity of muscle or heart phenotypes in dystrophic patients warrants further study.

## Additional file

Additional file 1:
**Figure S1.** TEM of GRAF1 tibialis anterior muscle. TEM (×2500 magnification) reveals T-tubule abnormalities in muscle from GRAF1-deficient mice. **Figure S2.** Graf1-depleted *mdx* mice exhibit increased muscle growth. Comparison of body mass and gross muscle size reveals that young adult Graf1-depleted *mdx* mice exhibit increased muscle growth relative to littermate controls. **Figure S3.** GRAF1/dystrophin- depleted tibialis anterior muscles contain smaller but more numerous fibers. Comparison of 6-month-old muscle cross-sectional areas reveals that GRAF1/dystrophin-depleted tibialis anterior muscles contain smaller but more numerous fibers than littermate controls. **Figure S4.** Adult GRAF1/dystrophin- depleted diaphragms contain more numerous fibers and exhibit signs of reduced regenerative capacity. Comparison of 6-month-old diaphragm muscle cross-sectional areas reveals that GRAF1/dystrophin-depleted tissue contains more numerous fibers and has less regenerative potential than littermate controls.

## Abbreviations

BAR: Bin–amphiphysin–Rvs
cDNA: complementary DNA
CSA: cross-sectional area
CTX: cardiotoxin
DAPI: 4′,6-diamidino-2-phenylindole
DGC: dystrophin glycoprotein complex
DM: differentiation media
DMD: Duchenne muscular dystrophy
EBD: Evans blue dye
EDTA: ethylenediaminetetraacetic acid
EF: ejection fraction
EHD1: EPS15 homology domain-containing 1
EHD2: EPS15 homology domain-containing 2
eMHC: embryonic myosin heavy chain
F-actin: filamentous actin
Fer1L5: ferlin-1-like 5
FS: fractional shortening
GAP: GTPase activating protein
GRAF1: GTPase regulator associated with focal adhesion kinase-1
GRMD: golden retriever muscular dystrophy
GT: gene trap
GTP: guanosine triphosphate
GTPase: guanosine triphosphatase
H&E: hematoxylin and eosin
IP: immunoprecipitation
ISO: isoproterenol
kDa: kilodalton
LV: left ventricle
LVID: left ventricle internal diameter
MCP-1: monocyte chemoattractant protein-1
MTC: Masson’s trichrome
NIS: non-immune serum
NTC: non-target control
PBS: phosphate-buffered saline
PCR: polymerase chain reaction
PH: pleckstrin homology
PM: plasma membrane
PS: phosphatidyl serine
RhoA: Ras homologue gene family, member A
RIPA: modified radioimmune precipitation assay
RT-PCR: reverse transcriptase polymerase chain reaction
sem: standard error of mean
sCK: serum creatine kinase
SDS-PAGE: sodium dodecyl sulfate-polyacrylamide gel electrophoresis
TBS: tris-buffered saline
TEM: transmission electron microscopy
WGA: wheat germ agglutinin
WT: wild type

## References

[1] Rybakova IN, Patel JR, Ervasti JM (2000). The dystrophin complex forms a mechanically strong link between the sarcolemma and costameric actin. J Cell Biol.

[2] Jung D, Yang B, Meyer J, Chamberlain JS, Campbell KP (1995). Identification and characterization of the dystrophin anchoring site on beta-dystroglycan. J Biol Chem.

[3] Koenig M, Monaco AP, Kunkel LM (1988). The complete sequence of dystrophin predicts a rod-shaped cytoskeletal protein. Cell.

[4] Ervasti JM, Ohlendieck K, Kahl SD, Gaver MG, Campbell KP (1990). Deficiency of a glycoprotein component of the dystrophin complex in dystrophic muscle. Nature.

[5] Bouter A, Gounou C, Berat R, Tan S, Gallois B, Granier T (2011). Annexin-A5 assembled into two-dimensional arrays promotes cell membrane repair. Nat Commun.

[6] Reddy A, Caler EV, Andrews NW (2001). Plasma membrane repair is mediated by Ca(2+)-regulated exocytosis of lysosomes. Cell.

[7] McNeil PL, Miyake K, Vogel SS (2003). The endomembrane requirement for cell surface repair. Proc Natl Acad Sci U S A.

[8] Idone V, Tam C, Goss JW, Toomre D, Pypaert M, Andrews NW (2008). Repair of injured plasma membrane by rapid Ca2+-dependent endocytosis. J Cell Biol.

[9] Bashir R, Britton S, Strachan T, Keers S, Vafiadaki E, Lako M (1998). A gene related to Caenorhabditis elegans spermatogenesis factor fer-1 is mutated in limb-girdle muscular dystrophy type 2B. Nat Genet.

[10] Liu J, Aoki M, Illa I, Wu C, Fardeau M, Angelini C (1998). Dysferlin, a novel skeletal muscle gene, is mutated in Miyoshi myopathy and limb girdle muscular dystrophy. Nat Genet.

[11] Wenzel K, Geier C, Qadri F, Hubner N, Schulz H, Erdmann B (2007). Dysfunction of dysferlin-deficient hearts. J Mol Med (Berl).

[12] Kuru S, Yasuma F, Wakayama T, Kimura S, Konagaya M, Aoki M (2004). A patient with limb girdle muscular dystrophy type 2B (LGMD2B) manifesting cardiomyopathy. Rinsho Shinkeigaku.

[13] Choi ER, Park SJ, Choe YH, Ryu DR, Chang SA, Choi JO (2010). Early detection of cardiac involvement in Miyoshi myopathy: 2D strain echocardiography and late gadolinium enhancement cardiovascular magnetic resonance. J Cardiovasc Magn Reson.

[14] Chase TH, Cox GA, Burzenski L, Foreman O, Shultz LD (2009). Dysferlin deficiency and the development of cardiomyopathy in a mouse model of limb-girdle muscular dystrophy 2B. Am J Pathol.

[15] Han R, Bansal D, Miyake K, Muniz VP, Weiss RM, McNeil PL (2007). Dysferlin-mediated membrane repair protects the heart from stress-induced left ventricular injury. J Clin Invest.

[16] Han R, Campbell KP (2007). Dysferlin and muscle membrane repair. Curr Opin Cell Biol.

[17] Bansal D, Miyake K, Vogel SS, Groh S, Chen CC, Williamson R (2003). Defective membrane repair in dysferlin-deficient muscular dystrophy. Nature.

[18] Demonbreun AR, Fahrenbach JP, Deveaux K, Earley JU, Pytel P, McNally EM (2011). Impaired muscle growth and response to insulin-like growth factor 1 in dysferlin-mediated muscular dystrophy. Hum Mol Genet.

[19] Posey AD, Demonbreun A, McNally EM (2011). Ferlin proteins in myoblast fusion and muscle growth. Curr Top Dev Biol.

[20] Martens S, Kozlov MM, McMahon HT (2007). How synaptotagmin promotes membrane fusion. Science.

[21] Matsuda C, Hayashi YK, Ogawa M, Aoki M, Murayama K, Nishino I (2001). The sarcolemmal proteins dysferlin and caveolin-3 interact in skeletal muscle. Hum Mol Genet.

[22] Cai C, Weisleder N, Ko JK, Komazaki S, Sunada Y, Nishi M (2009). Membrane repair defects in muscular dystrophy are linked to altered interaction between MG53, caveolin-3, and dysferlin. J Biol Chem.

[23] Evesson FJ, Peat RA, Lek A, Brilot F, Lo HP, Dale RC (2010). Reduced plasma membrane expression of dysferlin mutants is attributed to accelerated endocytosis via a syntaxin-4-associated pathway. J Biol Chem.

[24] Wallace GQ, McNally EM (2009). Mechanisms of muscle degeneration, regeneration, and repair in the muscular dystrophies. Annu Rev Physiol.

[25] Taylor JM, Hildebrand JD, Mack CP, Cox ME, Parsons JT (1998). Characterization of graf, the GTPase-activating protein for rho associated with focal adhesion kinase. Phosphorylation and possible regulation by mitogen-activated protein kinase. J Biol Chem.

[26] Doherty JT, Lenhart KC, Cameron MV, Mack CP, Conlon FL, Taylor JM (2011). Skeletal muscle differentiation and fusion are regulated by the BAR-containing Rho-GTPase-activating protein (Rho-GAP), GRAF1. J Biol Chem.

[27] Lenhart KC, Becherer AL, Li J, Xiao X, McNally EM, Mack CP (2014). GRAF1 promotes ferlin-dependent myoblast fusion. Dev Biol.

[28] Shin JH, Hakim CH, Zhang K, Duan D (2011). Genotyping mdx, mdx3cv, and mdx4cv mice by primer competition polymerase chain reaction. Muscle Nerve.

[29] Cheng Z, Dimichele LA, Rojas M, Vaziri C, Mack CP, Taylor JM. Focal adhesion kinase antagonizes doxorubicin cardiotoxicity via p21. J Mol Cell Cardiol. 2014;67:1–11. 10.1016/j.yjmcc.2013.12.002 PMC4237309 24342076

[30] Qiao C, Li J, Jiang J, Zhu X, Wang B, Li J (2008). Myostatin propeptide gene delivery by adeno-associated virus serotype 8 vectors enhances muscle growth and ameliorates dystrophic phenotypes in mdx mice. Hum Gene Ther.

[31] Louch WE, Sheehan KA, Wolska BM (2011). Methods in cardiomyocyte isolation, culture, and gene transfer. J Mol Cell Cardiol.

[32] DiFranco M, Quinonez M, Capote J, Vergara J. DNA transfection of mammalian skeletal muscles using in vivo electroporation. J Vis Exp. 2009;32. 10.3791/1520 PMC2793085 19841615

[33] Swaggart KA, Demonbreun AR, Vo AH, Swanson KE, Kim EY, Fahrenbach JP (2014). Annexin A6 modifies muscular dystrophy by mediating sarcolemmal repair. Proc Natl Acad Sci U S A.

[34] Cai C, Masumiya H, Weisleder N, Matsuda N, Nishi M, Hwang M (2009). MG53 nucleates assembly of cell membrane repair machinery. Nat Cell Biol.

[35] Lennon NJ, Kho A, Bacskai BJ, Perlmutter SL, Hyman BT, Brown RH (2003). Dysferlin interacts with annexins A1 and A2 and mediates sarcolemmal wound-healing. J Biol Chem.

[36] Huang Y, Laval SH, van Remoortere A, Baudier J, Benaud C, Anderson LV (2007). AHNAK, a novel component of the dysferlin protein complex, redistributes to the cytoplasm with dysferlin during skeletal muscle regeneration. Faseb J.

[37] Gerke V, Creutz CE, Moss SE (2005). Annexins: linking Ca2+ signalling to membrane dynamics. Nat Rev Mol Cell Biol.

[38] Rezvanpour A, Santamaria-Kisiel L, Shaw GS (2011). The S100A10-annexin A2 complex provides a novel asymmetric platform for membrane repair. J Biol Chem.

[39] Zacharias U, Purfurst B, Schowel V, Morano I, Spuler S, Haase H (2011). Ahnak1 abnormally localizes in muscular dystrophies and contributes to muscle vesicle release. J Muscle Res Cell Motil.

[40] Haase H, Alvarez J, Petzhold D, Doller A, Behlke J, Erdmann J (2005). Ahnak is critical for cardiac Ca(V)1.2 calcium channel function and its beta-adrenergic regulation. Faseb J.

[41] Hwang M, Ko JK, Weisleder N, Takeshima H, Ma J (2011). Redox-dependent oligomerization through a leucine zipper motif is essential for MG53-mediated cell membrane repair. Am J Physiol Cell Physiol.

[42] Draeger A, Monastyrskaya K, Babiychuk EB (2011). Plasma membrane repair and cellular damage control: the annexin survival kit. Biochem Pharmacol.

[43] Selcen D, Stilling G, Engel AG (2001). The earliest pathologic alterations in dysferlinopathy. Neurology.

[44] Kerr JP, Ziman AP, Mueller AL, Muriel JM, Kleinhans-Welte E, Gumerson JD (2014). Dysferlin stabilizes stress-induced Ca2+ signaling in the transverse tubule membrane. Proc Natl Acad Sci U S A.

[45] Klinge L, Laval S, Keers S, Haldane F, Straub V, Barresi R (2007). From T-tubule to sarcolemma: damage-induced dysferlin translocation in early myogenesis. Faseb J.

[46] Klinge L, Harris J, Sewry C, Charlton R, Anderson L, Laval S (2010). Dysferlin associates with the developing T-tubule system in rodent and human skeletal muscle. Muscle Nerve.

[47] Chiu YH, Hornsey MA, Klinge L, Jorgensen LH, Laval SH, Charlton R (2009). Attenuated muscle regeneration is a key factor in dysferlin-deficient muscular dystrophy. Hum Mol Genet.

[48] Han R, Rader EP, Levy JR, Bansal D, Campbell KP (2011). Dystrophin deficiency exacerbates skeletal muscle pathology in dysferlin-null mice. Skelet Muscle.

[49] Illa I, Serrano-Munuera C, Gallardo E, Lasa A, Rojas-Garcia R, Palmer J (2001). Distal anterior compartment myopathy: a dysferlin mutation causing a new muscular dystrophy phenotype. Ann Neurol.

[50] Nguyen K, Bassez G, Krahn M, Bernard R, Laforet P, Labelle V (2007). Phenotypic study in 40 patients with dysferlin gene mutations: high frequency of atypical phenotypes. Arch Neurol.

[51] Roche JA, Lovering RM, Bloch RJ (2008). Impaired recovery of dysferlin-null skeletal muscle after contraction-induced injury in vivo. Neuroreport.

[52] Kornegay JN, Bogan JR, Bogan DJ, Childers MK, Grange RW (2011). Golden retriever muscular dystrophy (GRMD): Developing and maintaining a colony and physiological functional measurements. Methods Mol Biol.

[53] Cai B, Xie S, Caplan S, Naslavsky N (2014). GRAF1 forms a complex with MICAL-L1 and EHD1 to cooperate in tubular recycling endosome vesiculation. Front Cell Dev Biol.

[54] Posey AD, Pytel P, Gardikiotes K, Demonbreun AR, Rainey M, George M (2011). Endocytic Recycling Proteins EHD1 and EHD2 Interact with Fer-1-like-5 (Fer1L5) and Mediate Myoblast Fusion. J Biol Chem.

[55] Durieux AC, Vignaud A, Prudhon B, Viou MT, Beuvin M, Vassilopoulos S (2010). A centronuclear myopathy-dynamin 2 mutation impairs skeletal muscle structure and function in mice. Hum Mol Genet.

[56] Piccolo F, Moore SA, Ford GC, Campbell KP (2000). Intracellular accumulation and reduced sarcolemmal expression of dysferlin in limb—girdle muscular dystrophies. Ann Neurol.

[57] Barresi V, Ragusa A, Fichera M, Musso N, Castiglia L, Rappazzo G (2010). Decreased expression of GRAF1/OPHN-1-L in the X-linked alpha thalassemia mental retardation syndrome. BMC Med Genomics.

[58] Zohrabian VM, Nandu H, Gulati N, Khitrov G, Zhao C, Mohan A (2007). Gene expression profiling of metastatic brain cancer. Oncol Rep.

[59] Bojesen SE, Ammerpohl O, Weinhausl A, Haas OA, Mettal H, Bohle RM (2006). Characterisation of the GRAF gene promoter and its methylation in patients with acute myeloid leukaemia and myelodysplastic syndrome. Br J Cancer.

[60] Borkhardt A, Bojesen S, Haas OA, Fuchs U, Bartelheimer D, Loncarevic IF (2000). The human GRAF gene is fused to MLL in a unique t(5;11)(q31;q23) and both alleles are disrupted in three cases of myelodysplastic syndrome/acute myeloid leukemia with a deletion 5q. Proc Natl Acad Sci U S A.

[61] Wilda M, Perez AV, Bruch J, Woessmann W, Metzler M, Fuchs U (2005). Use of MLL/GRAF fusion mRNA for measurement of minimal residual disease during chemotherapy in an infant with acute monoblastic leukemia (AML-M5). Genes Chromosomes Cancer.

