# Supplementary material for: GRAF1 deficiency blunts sarcolemmal injury repair and exacerbates cardiac and skeletal muscle pathology in dystrophin-deficient mice
Source: Skelet Muscle. 2015 Aug 21;5:27. doi: 10.1186/s13395-015-0054-6 (PMC4546166; doi:10.1186/s13395-015-0054-6)
Supplement: Additional file 1: — Figure S1. TEM of GRAF1 tibialis anterior muscle. TEM (×2500 magnification) reveals T-tubule abnormalities in muscle from GRAF1-deficient mice. Figure S2. Graf1-depleted mdx mice exhibit increased muscle growth. Comparison of body mass and gross muscle size reveals that young adult Graf1-depleted mdx mice exhibit increased muscle growth relative to littermate controls. Figure S3. GRAF1/dystrophin- depleted tibialis anterior muscles contain smaller but more numerous fibers. Comparison of 6-month-old muscle cross-sectional areas reveals that GRAF1/dystrophin-depleted tibialis anterior muscles contain smaller but more numerous fibers than littermate controls. Figure S4. Adult GRAF1/dystrophin- depleted diaphragms contain more numerous fibers and exhibit signs of reduced regenerative capacity. Comparison of 6-month-old diaphragm muscle cross-sectional areas reveals that GRAF1/dystrophin-depleted tissue contains more numerous fibers and has less regenerative potential than littermate controls. [file 13395_2015_54_MOESM1_ESM.pdf]

Supplemental Data File for Lenhart et. al. contains 4 figures with accompanying legends

Supplemental Figure Legends:

Supplemental Figure 1. TEM (2500X magnification) reveals T-tubule abnormalities in muscle from GRAF1-deficient mice.

Supplemental Figure 2: Graf1-depleted *mdx* mice exhibit increased muscle growth. (a) Representative images of 6 month old male mice with indicated genotypes. Note pronounced size increase of body (*top panels*), forelimbs (*middle panels*), and hindlimbs (*bottom panels*) from $Graf1^{Gt/Gt}$;$X^{mdx}Y$ mice compared to age-matched controls. **(b)** Comparison of gastrocnemius muscles isolated from mice in panel A reveal marked muscle growth in $Graf1^{Gt/Gt}$;$X^{mdx}Y$ mice. **(c)** Average body mass of 6 month old male mice. Data are represented as mean ± s.e.m. (**p*<0.005; **p*<0.05; *N*=5 mice per genotype).

Supplemental Figure 3. GRAF1/dystrophin- depleted tibialis anterior muscles contain smaller but more numerous fibers (a) Representative cross-sectional images of tibialis anterior muscle from 6 month male mice with indicated genotypes. Wheat germ agglutinin (WGA, red) demarcates myofiber boundaries. Nuclei are counterstained with DAPI (blue). Note increased fiber size variability in double deficient muscles. **(a, bottom)** Frequency histogram demonstrates myofiber distribution by cross-sectional area (CSA). **(b)** Average myofiber CSA (**p*<0.001; *n*=350 myofibers per mouse; *N*=5 mice per genotype). **(c)** Average myofiber number per 0.5 mm^2 (**p*<0.05; *N*=5 mice per genotype). Data are represented as mean ± s.e.m. (**d**) Graphical representation of the percentage of regenerating myofibers in the muscle (n.s.=no significance*;* *n*=300 myofibers per mouse; *N*=5 mice per genotype). Data are represented as mean ± s.e.m. Scale bars=100 µm.

Supplemental Figure 4. Adult GRAF1/dystrophin- depleted diaphragms contain more numerous fibers and exhibit signs of reduced regenerative capacity (**a**) Quantification of total number of diaphragm myofibers from 6 month old male mice (*N*=3-5 mice per genotype). **(b)** Diaphragm cross sections from 6 month old male mice with indicated genotypes immunostained with eMHC (green) to demarcate regenerative fibers. Note marked reduction in regenerating fibers in the *mdx*/Graf1-deficient muscles (*arrows*). Scale bars=20 µm. (**c**) Percentage of regenerating myofibers in diaphragm muscles from 6 month old male mice as assessed by central nucleation (*n*=1,000 myofibers per mouse; *N*=3-5 mice per genotype). n.s.=not significant. Data are represented as mean ± s.e.m.

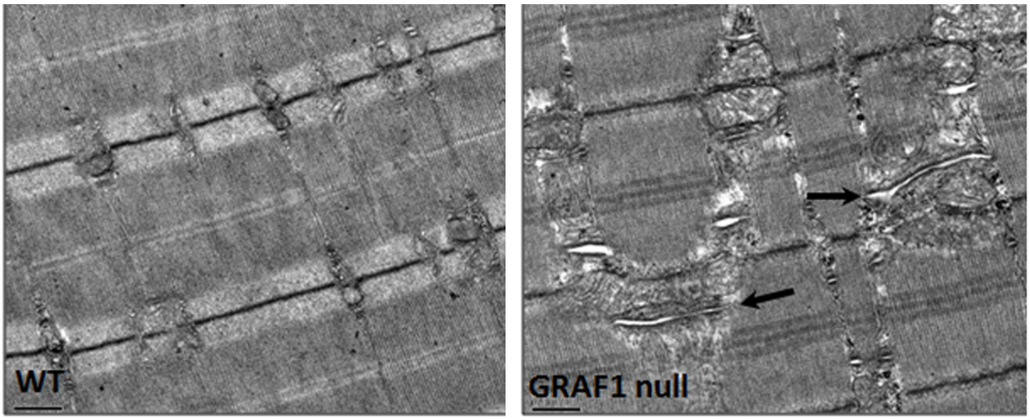

Lenhart et. al. Supplemental Figure 1

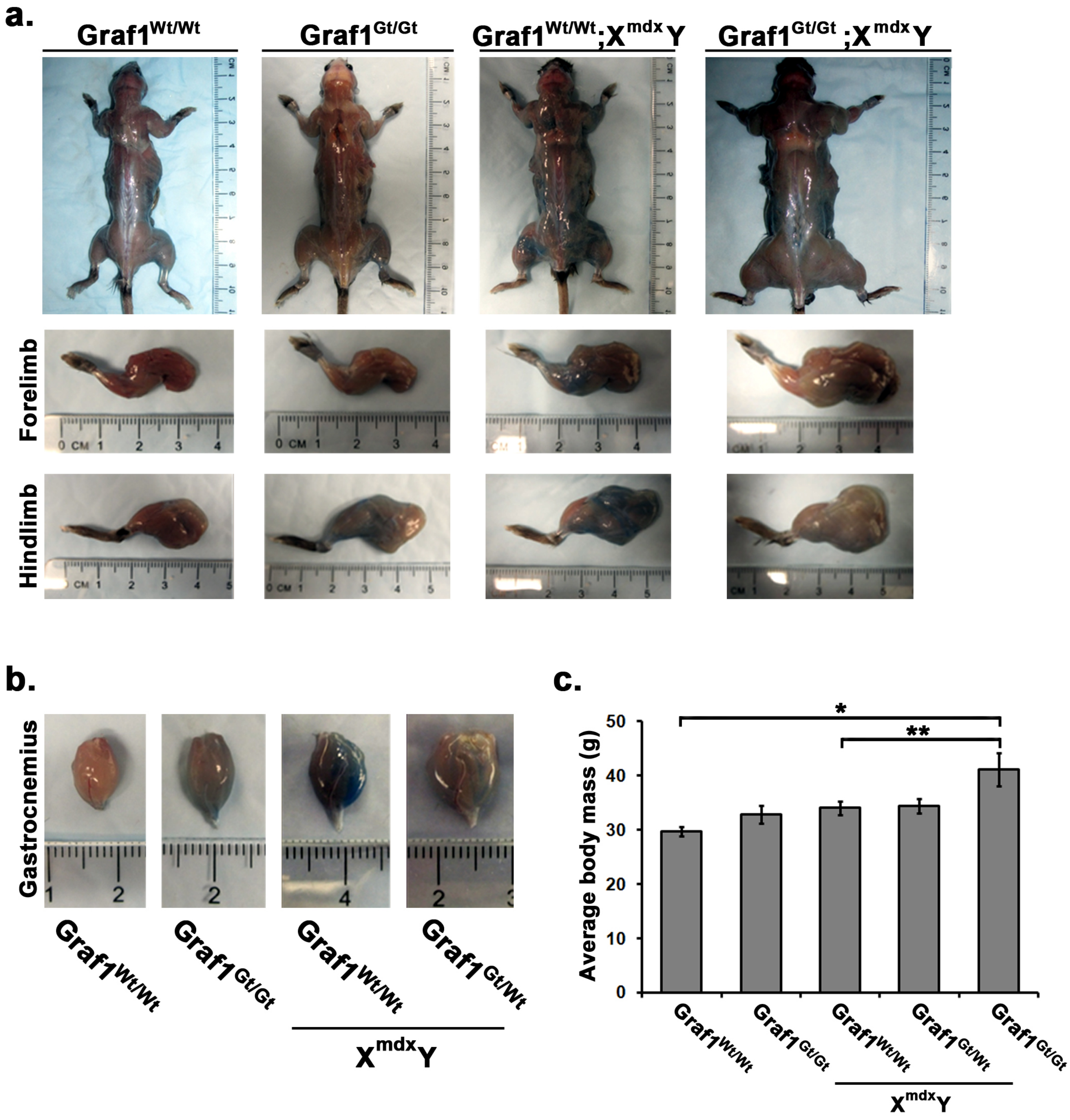

Lenhart et. al. Supplemental Figure 2

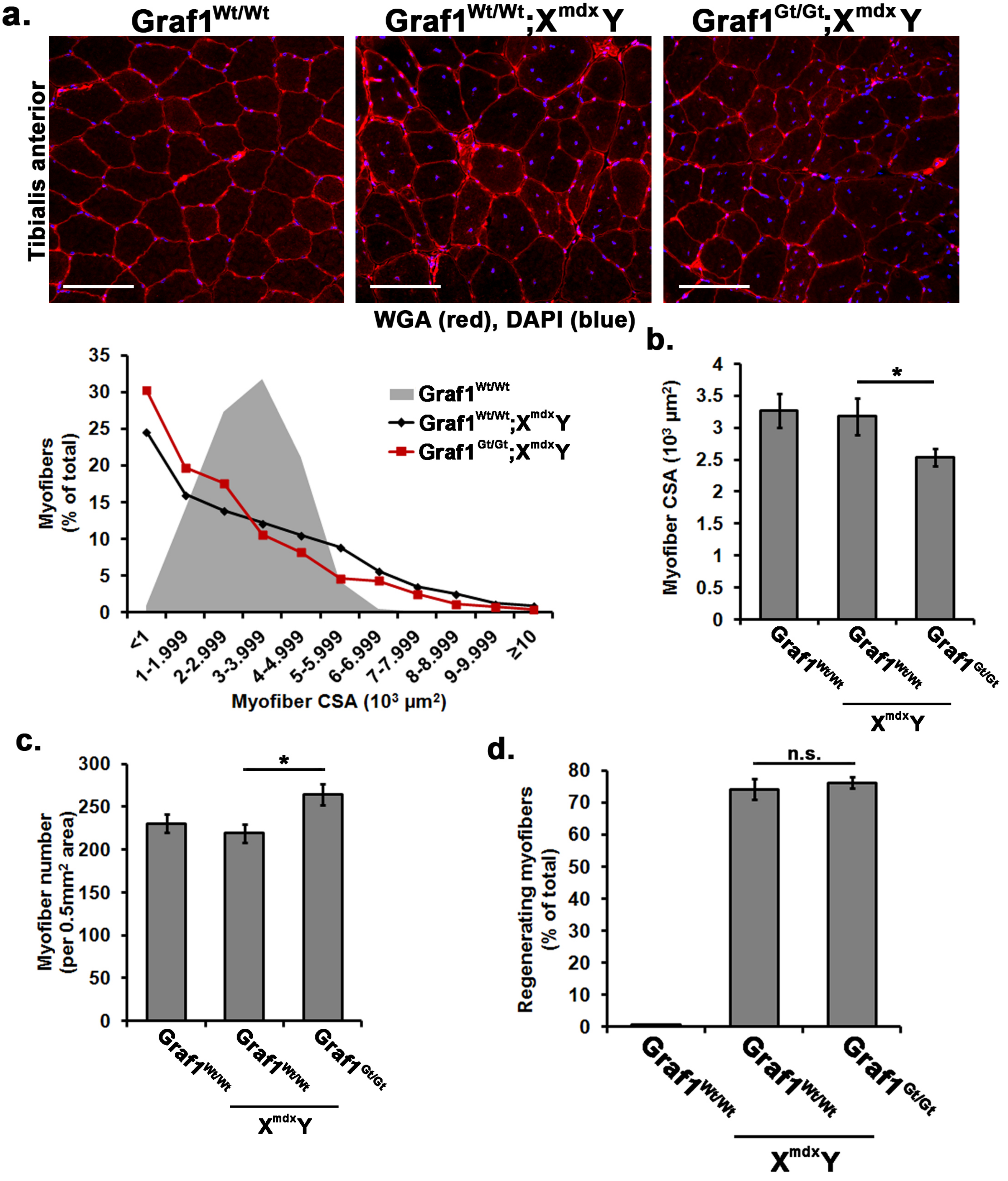

Lenhart et.al. Supplemental Fig 3

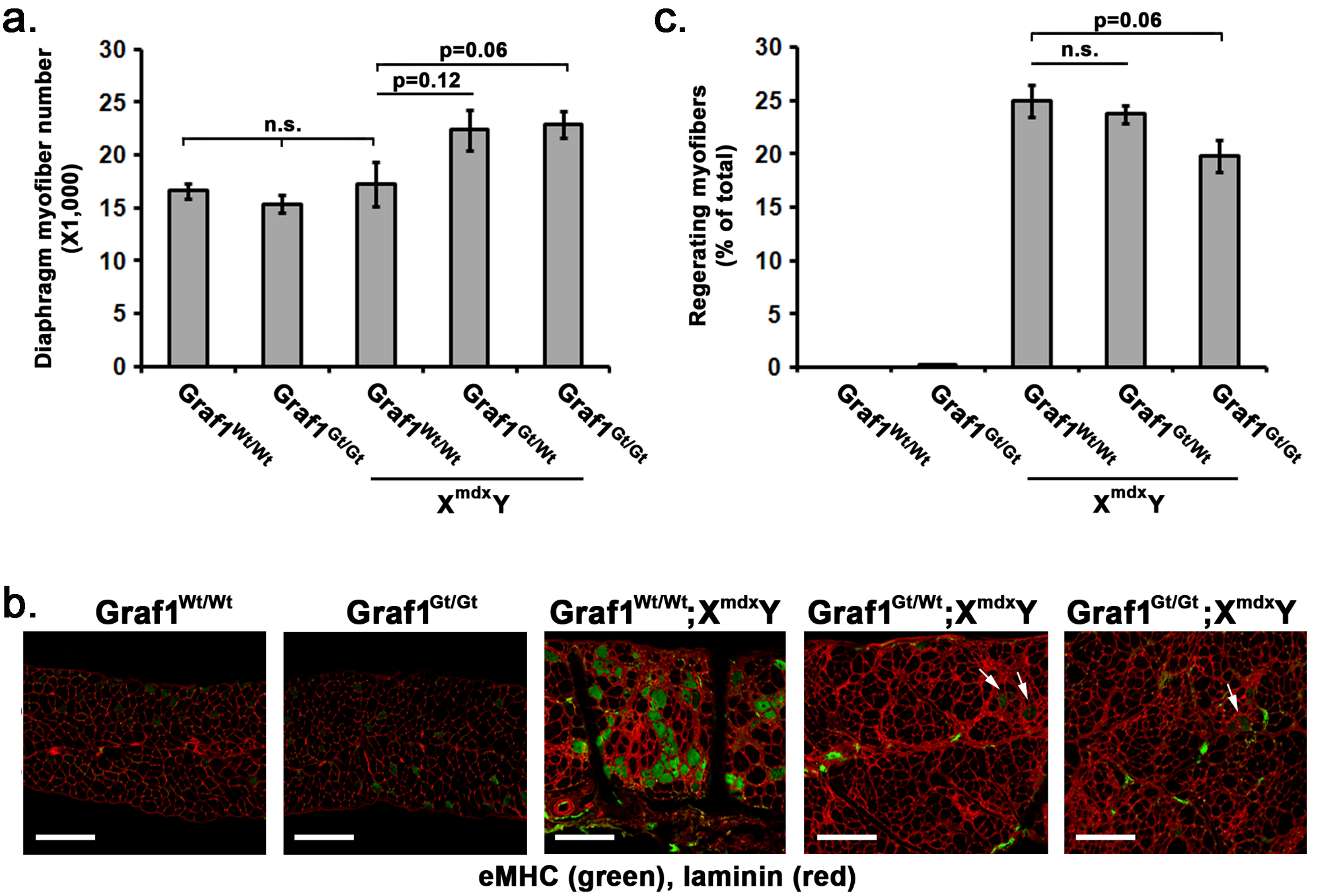

Lenhart et. al. Supplemental Figure 4
